# Vapor Adsorption Measurements with Two‐Dimensional Membranes

**DOI:** 10.1002/cphc.202100732

**Published:** 2021-11-30

**Authors:** Petr Dementyev, Neita Khayya, Jakob Kreie, Armin Gölzhäuser

**Affiliations:** ^1^ Faculty of Physics Bielefeld University Universitätsstr. 25 33615 Bielefeld Germany

**Keywords:** two-dimensional membranes, vapor permeation, vapor adsorption, infrared reflection absorption spectroscopy, azeotropes

## Abstract

Two‐dimensional (2D) membranes display extraordinary mass transfer properties, in particular for the permeation of gaseous substances. Their ultimate thickness not only ensures the shortest diffusion pathways, but also makes the membrane surface play a significant role in accommodating and guiding the permeating molecules. As saturated vapors of water and organic solvents are often observed to pass 2D membranes faster than inert gases, condensation is believed to be responsible for surface‐mediated transport. Here, we present a spectroscopic experiment to probe adsorption of condensable species on 2D membranes under realistic conditions. Polarization‐modulation infrared reflection absorption spectroscopy (PM IRAS) is coupled with a reaction chamber and a vacuum system to control the vaporous environments. The measurements are demonstrated to yield quantitative information on the amount of adsorbates onto supported 2D layers. As a case study, the azeotropic mixture of water and propanol is revealed to maintain its molar composition upon interaction with carbon nanomembranes.

## Graphical abstract

**A new experiment is introduced** to study the interaction of vaporous substances with 2D membranes. Infrared spectroscopy is shown to provide knowledge on the amount of adsorbed species under varied conditions. Water vapor mixed with propanol is found to condense on the membrane surface with the same molar proportions as in the original feed mixture.

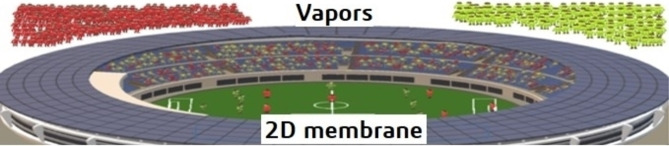

## Introduction

Driven initially by perforation of single‐layer materials,^[1]^ two‐dimensional (2D) membranes keep drawing attention as there emerge different types of planar nanostructures with inherent porosity. These include 2D polymers such as covalent organic frameworks (COFs),^[2]^ tetrahedral bilayer oxides including 2D SiO_2_,^[3]^ carbon nanomembranes (CNMs),^[4]^ etc. Unlike artificially pierced graphene, intrinsically porous 2D membranes exhibit monodisperse or very narrow size distributions and high areal densities of nanoscopic pores. Recent advances in synthetic methods enabled the materials to be obtained on a centimeter scale,^[5]^ and model mass transfer studies with free‐standing membranes revealed intriguing performance characteristics.^[6]^ It was observed that gas permeation across 2D membranes depends not only on the relative dimensions of molecules and pores, but also on the ability of gaseous species to interact with the membrane surface.^[7]^ In accordance with the escape problem of statistical physics,^[8]^ a particle diffusing along a plane is much more likely to find an exit compared to a particle traveling in three dimensions. Hence, adsorption on 2D materials appears to be an important channel to populate the near‐membrane area and promote the surface‐mediated transport in addition to the free molecular flow.

While ordinary gases hardly adsorb under ambient temperature, 2D membranes were shown to allow for enhanced permeation rates towards vaporous substances.^[9]^ The selectivity over noble and atmospheric gases achieved with CNMs was speculated to stem from facile molecular condensation upon exposure to saturated vapors. As vapor permeation itself is technologically relevant for dehydration of organic solvents and production of high‐grade biofuels,^[10]^ room‐temperature experiments with water, alcohols and their mixtures proved the potential of CNMs in breaking azeotropes.^[11]^ However, there were several interesting phenomena seen that are immediately related to vapor adsorption on 2D membranes.^[12]^ On the one hand, the permeation rate of water mixed with n‐propanol was found to be almost the same as for pure water indicating a similar amount of adsorbates on the membrane surface. On the other hand, double‐layer CNMs exposed to the mixture exhibited a ten times lower flow rate for water which was attributed to the pore blockage by dissolved alcohol molecules.^[11]^ The effect of concentration was also evident upon mixing water vapor with isopropanol, and the greater the molar fraction of the alcohol, the lower the transmembrane water flux.^[12]^ It appeared that water and alcohol vapors were able to adsorb on the membrane surface simultaneously, but the exact proportions in the condensed phase remained elusive.

The concept of Adsorption Controlled Permeation (ACP) was established for describing vapor permeation rates in inherent 2D membranes with the pore density exceeding 10^12^ cm^−2^.[^9^, ^12^] The latter number means that adjacent pores are a few nm away from each other, and the spacing is available for vapor molecules to adsorb. The membrane pores are considered as a sink for the adsorbates whereas the transmembrane flux *F* is expressed in terms of heterogeneous reaction kinetics:

F=k·θ

where *k* is the effective first‐order rate constant, and *θ* is the absolute surface coverage of adsorbed molecules. The rate constant is a function of the molecular size and accounts for sieving properties of the membrane. In turn, the coverage is a thermodynamic characteristic and obeys adsorption isotherm equations. The ACP formalism proved to explain both the drastic difference between inert gases and vapors as well as the pronounced non‐linear dependence of the permeance on pressure.^[9]^ Despite the prospects of the ACP methodology to help understand mechanisms of the interfacial transport, the approach needs to be justified by direct adsorption studies.

In this work, we design a spectroscopic experiment to explore vapor condensation on supported 2D membranes under conditions identical to the permeation measurements. Given the working pressure of up to several hundred mbar, polarization‐modulation infrared reflection absorption spectroscopy (PM IRAS) is selected as a suitable surface‐sensitive technique to probe the amount of adsorbed molecules on flat substrates.^[13]^ In order to precisely regulate gaseous surroundings, a vacuum system is built on the basis of a commercial reaction chamber compatible with PM IRAS. We demonstrate the instrument operation with CNMs exposed to heavy water, n‐propanol as well as to their azeotropic mixture with the mole ratio of 3 : 2. These substances are chosen as a convenient model system that was used before for probing molecular separation. All the vapors are shown to readily adsorb on the membrane surface in dependence of their relative pressure. More importantly, the vaporous mixture is found to preserve its molar composition upon condensation confirming the previous permeation studies. The new experiment appears to provide valuable information on gas‐surface interactions that take place upon the ACP processes in 2D membranes.

## Results and Discussion

### System Design and Operation

**Figure 1:**
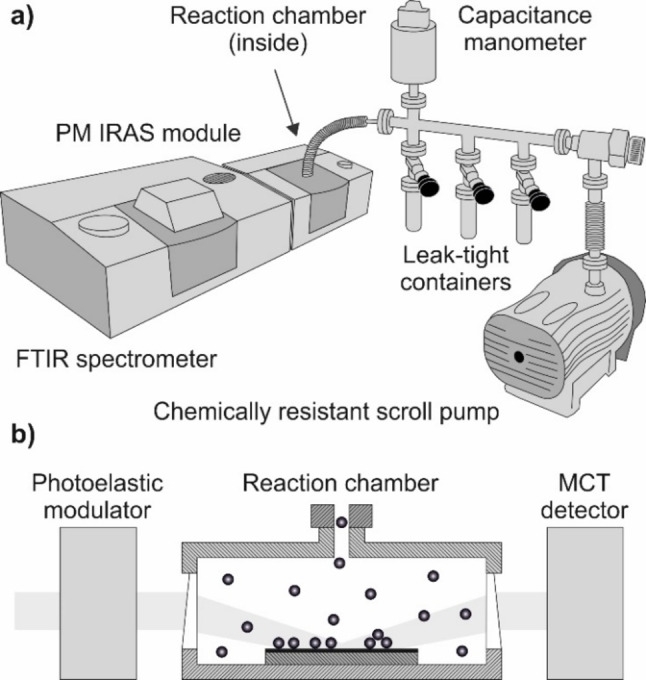
Schematic of vapor adsorption measurements with 2D membranes: a) system overview; b) experimental configuration.

As the ACP measurements are done with microscopic free‐standing 2D membranes,^[9]^ it is highly challenging to implement any kind of spectroscopic characterization directly to the permeation equipment. Instead, we propose an auxiliary setup to reproduce the experimental conditions, but with macroscopic 2D layers supported by flat substrates. To ensure the surface‐sensitivity and avoid the interference with gaseous species, PM IRAS measurements are carried out in a compact reaction chamber connected to a gas supply line. Figure 1 illustrates the technical arrangement and the principle of the vapor adsorption experiment.

The Refractor^TM^ reactor is placed into the polarization‐modulation unit of the Fourier‐transform infrared (FTIR) spectrometer and connected via the flexible metal tubing to the stainless‐steel feed reservoir. The latter is equipped with the capacitance manometer and carries three leak‐tight containers with liquid substances. The scroll pump is used to evacuate the system to a base pressure of 10^−3^ mbar as well as to remove air from the vapors. As opposed to the conventional freeze‐pump‐thaw method, degassing is done by opening a liquid container to the feed reservoir when the angle valve to the pump is closed. Due to a great volume difference, air from the container is mostly redistributed to the reaction chamber and the feed reservoir that are pumped out after the container is sealed again. The procedure is repeated a few times before the vapor pressure reaches a constant value meaning a negligible fraction of residual gas molecules. The outgassed vapors are let to the reaction chamber via dosing valves, so that the pressure in the system can be finely adjusted up to the saturation points as monitored by the manometer. Similar to the permeation experiments, adsorption measurements are performed in the isobaric regime once the vapor pressure is fixed at a certain value. Following the spectral acquisition, the substances are discharged to the chemically resistant pump in order to clean up the reaction chamber and the 2D membrane.

To enable the light reflection, 2D membranes need to be positioned on a conducting surface. This is achieved by the transfer onto appropriate supports or occurs naturally during the synthesis as many 2D materials are grown on metals, e. g. bilayer oxides and CNMs. The Refractor^TM^ chamber can host horizontally mounted samples of up to 4 cm in length and 2.5 cm wide. The linearly polarized infrared beam passes first the photoelastic modulator and is then refracted to the supported membrane by a wedged ZnSe window (Figure 1b). The incidence angle is set at 75°, and the reflected light is directed to the detector through another ZnSe window. The in‐line optical configuration provides the shortest beam path through the vaporous atmosphere and least energy losses associated with scattering and absorption. The mercury cadmium telluride (MCT) photodetector is cooled down by liquid nitrogen, whereas the 2D membrane is held at room temperature to replicate the ACP environments. There is also a built‐in heater available in the reaction chamber to elevate the membrane temperature which can be used to study thermodynamics of vapor condensation.

The PM IRAS measurements allow for excluding the gas‐phase contribution from the total absorption and yielding vibrational spectra of the adsorbed molecules and the 2D material solely. More specifically, the photoelastic modulator alternates the beam polarization between parallel (*s*‐polarization) and perpendicular (*p*‐polarization) to the membrane surface. As the light reflection from a metal surface leads to a phase shift, the *s*‐polarized electric field is canceled out in vicinity of the surface whereas an enhanced standing wave is formed for the *p*‐polarization.^[13]^ Therefore, molecular adsorbates interact with the *p*‐polarized light, and the metal‐surface selection rules apply that suggest vibrations with an upright dynamic dipole to be visible in IRAS.^[14]^ Because gaseous species equally absorb both polarizations, the surface sensitivity arises in the differential reflectance that is measured and processed by the instrument electronics. The resulting PM IRAS output is affected by the demodulation treatment and normally does not comply with the Beer‐Lambert law.^[13]^ Although a calibration procedure was developed for converting PM IRAS data into absorption spectra,^[15]^ in this study we rely on a simplified algorithm to quantify the measurements. In a first approximation,^[13]^ direct PM IRAS signals are normalized by background spectra that are recorded in vacuum upon continuous pumping. Baseline correction is then performed manually to obtain integral spectral intensities.

### Vapor Adsorption on CNMs

**Figure 2:**
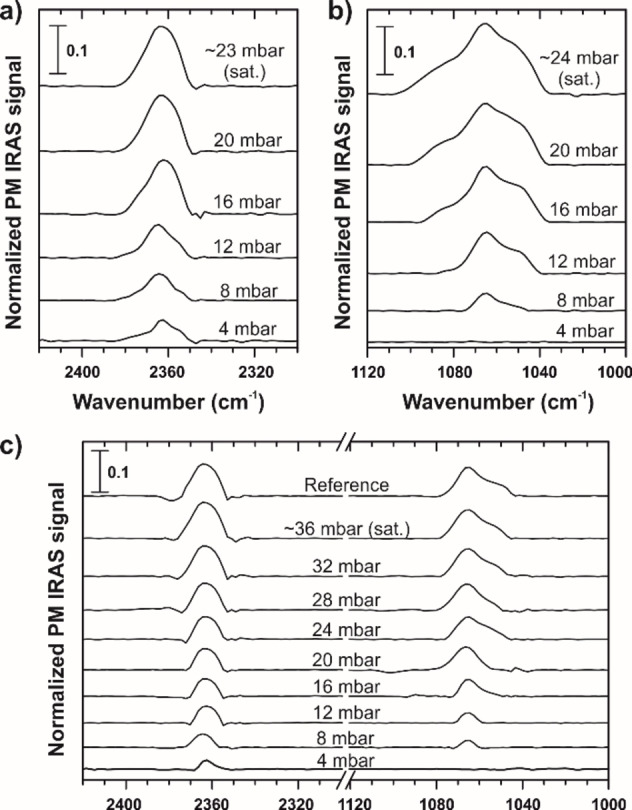
Exemplary PM IRAS spectra of vapor molecules adsorbed on CNMs as a function of pressure: a) heavy water; b) n‐propanol; c) azeotropic mixture of heavy water and n‐propanol with the mole ratio 3 : 2 respectively.

To test the newly constructed system, we studied the interaction of heavy water and n‐propanol with CNMs supported by native thin‐film gold substrates. The as‐prepared membrane was located in the reaction chamber and subjected to the pure vapors as well as to their mixture in azeotropic composition, i. e. the mixture with constant mole fractions of the components in the liquid and the gas phases.^[11]^ Unlike the previous permeation studies,^[11]^ the azeotrope was prepared *ex situ* by mixing liquid water and propanol in the molar proportion of 3 : 2 respectively.^[16]^ The solution was stored and handled in a leak‐tight container similar to the individual compounds, whereas each vapor was separately dosed to the reaction chamber to gradually change the pressure. The total pressure of the mixture at saturation was 1.5 times higher compared to saturated vapors of the pure substances, and the partial pressure of the components was determined from the mole ratio. Figure 2 illustrates the PM IRAS spectra of the adsorbates on the 2D membrane obtained upon incremental increase of the feed pressure.

The most pronounced absorption band for heavy water appears at around 2365 cm^−1^ and can be assigned to hydrogen‐bonded OD stretching modes. The spectral intensity clearly grows with the vapor pressure indicating the surface uptake of water molecules (Figure 2a). However, the peak position seems to be red‐shifted compared to bulk D_2_O as the band is typically centered at 2480 cm^−1^ for both liquid‐phase and adsorption experiments.[^17^, ^18^] It is worth noting that the IRAS data on heavy water were predominantly taken in ultrahigh vacuum at cryogenic temperatures which favors the formation of ice‐like overlayers.[^19^, ^20^] In turn, condensation of vaporous H_2_O under atmospheric pressure was studied with transmission FTIR as a function of relative humidity revealing two distinct absorption features.^[21]^ While the peak at 3400 cm^−1^ was identical to that of bulk water, the low‐frequency band at 3200 cm^−1^ was attributed to small water clusters with a different hydrogen‐bonding network. This was consistent with the microscopic observation of water nanodroplets formed at surface defects under ambient atmosphere.^[22]^ Recently, D_2_O clusters were isolated in rare‐gas matrices, and their IRAS fingerprints were found to change with the cluster size.^[23]^ As the hydrogen‐bonded OD stretching vibrations in the clusters appeared red‐shifted, we interpret our spectra to be associated with D_2_O agglomerates forming at the nanostructured surface of CNMs.

The characteristic absorption band for n‐propanol is seen at 1065 cm^−1^ (Figure 2b) that agrees well with vapor‐ and liquid‐phase measurements.[^24^, ^25^] The band is commonly assigned to CO stretching modes and seems not to change upon condensation. As evident, the peak intensity steadily goes up with pressure confirming the alcohol adsorption on CNMs and our previous conclusions on the membrane selectivity.^[11]^ This means that in contrast to methanol and ethanol vapors, the permeation of n‐propanol in CNMs was indeed hindered by its molecular size despite the abundance of adsorbates. Interestingly, the spectral feature also emerges when the membrane is exposed to the mixture of the alcohol with water vapor. One can see in Figure 2c that both heavy water and n‐propanol adsorb simultaneously, and their amount on the surface constantly increases as the vapor pressure approaches saturation. It appears that neither the peak positions nor their shapes are affected by the presence of the other substance. At first sight the absorption bands of water and the alcohol look independent from each other, but their intensity is different with respect to the spectra collected with the pure vapors (Figure 2a,b). To figure out whether the homogenous vapor mixture is separated on the membrane surface or not, we analyzed the integral peak intensities.

**Figure 3:**
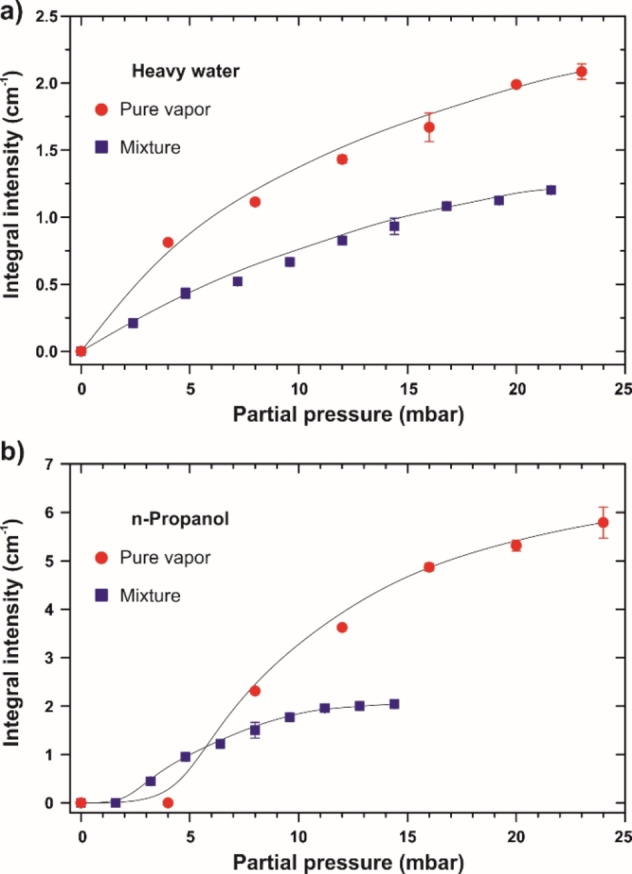
Comparison of integrated PM IRAS intensities in pure vapors and the mixture. a) Integrated spectral intensity for adsorbed heavy water. b) Integrated spectral intensity for adsorbed n‐propanol. The partial pressure of heavy water and n‐propanol in the mixture experiments is determined from the total vapor pressure with the molar fractions 0.6 and 0.4 respectively. Data points are average values over three measurements. Solid lines are to guide the eye.

First, the PM IRAS signals are plotted versus the partial pressure of the vapors (Figure 3). While for pure substances it is simply the pressure as determined by the manometer, the molar fractions 0.6 and 0.4 are used to calculate the partial pressure of heavy water and n‐propanol from the total pressure of the vaporous mixture. Given the saturation vapor pressure of ∼36 mbar for the azeotrope, ∼23 mbar for heavy water, and ∼24 mbar for n‐propanol, the same partial pressure for a given component corresponds to different relative pressures when divided by the saturation points. For example, 5 mbar of pure n‐propanol translates into its relative pressure of ∼0.21, whereas 5 mbar of n‐propanol in the mixture means the relative pressure to be ∼0.35. This explains why at low partial pressure the amount of adsorbed alcohol appears to be greater for the mixture than in the pure vapor. Indeed, it is the relative pressure that is an independent variable in adsorption isotherm expressions for condensable species such as Brunauer‐Emmett‐Teller (BET) theory.^[26]^

Although the shape of the resulting curves is similar for the individual compounds and the azeotrope, the maximum intensity of both heavy water and n‐propanol is reduced in the latter case. The amount of adsorbed water on CNMs exposed to the mixture appears to be as much as 60 % of that under saturated D_2_O vapor. On the contrary, there is as little as 35 % of n‐propanol adsorbed from the azeotrope compared to the alcohol vapor alone. The relations are striking in a way that they closely remind the molar proportions in the mixture. Assuming that pure vapors occupy the same adsorption sites on the membrane surface, the decrease in the spectral intensity may indicate competitive adsorption. On the other hand, the azeotropic nature of the mixture implies the substances to be condensed together, and the reduction may be reflective of the dilution. Therefore, we performed a control experiment with a liquid mixture of heavy water and n‐propanol to acquire reference PM IRAS signals (Figure 2c). To this end, the reaction chamber was vented and a 5‐μL droplet of the 3 : 2 mol. mixture was cast onto the CNM sample followed by recording the spectra in air upon liquid evaporation. Despite the beam attenuation by the macroscopic droplet, it turned out possible to capture the moment when the solution no longer interfered with the IRAS measurements whereas there were still liquefied molecules present on the membrane.

**Figure 4:**
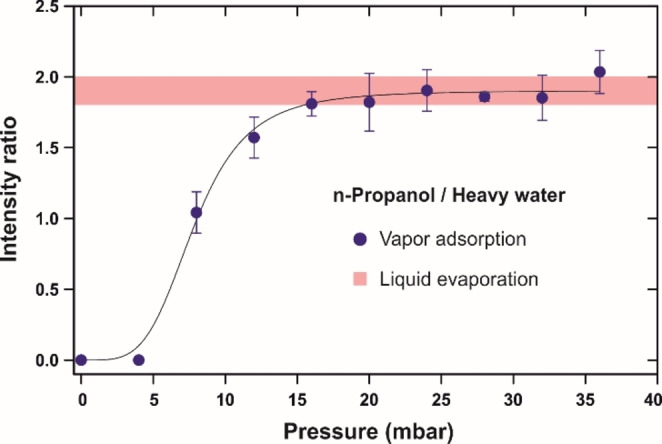
Ratio of integrated PM IRAS signals for n‐propanol and heavy water in the experiments with the azeotropic mixture (0.6 mol. D_2_O, 0.4 mol. C_3_H_7_OH). Dark circles were obtained upon vapor adsorption as a function of the total pressure. Shaded area represents the scatter over four measurements upon evaporation of the reference liquid mixture (0.6 mol. D_2_O, 0.4 mol. C_3_H_7_OH). Data points are average values over three measurements, and the error bars are standard deviation. Solid line is for guiding the eye.

Figure 4 shows the spectral intensity of n‐propanol divided by that of heavy water in the experiments with the vaporous azeotrope. This serves as an indirect measure of the molar proportions in the adsorbed phase, and the data points are plotted as a function of the total pressure of the mixture. Despite the constant molar composition 3 : 2 in the gas phase, the ratio appears to change at low pressure which is explained by preferential adsorption of water molecules. As evident from the spectra in Figure 2c and the integrals in Figure 3, there is not much alcohol seen at 4 and 8 mbar. However, the curve levels off after 15 mbar, and the intensity ratio comes to a constant value that matches very well to the reference shaded area obtained with the liquid azeotrope. This proves that condensation of the vaporous mixture occurs concertedly, i. e. the adsorbed phase also represents a homogeneous water‐propanol solution. It is likely that water molecules interact stronger with the membrane surface and start adsorbing first, but the alcohol catches up at higher pressures and compensates the azeotropic composition. In other words, the molar fractions of water and the alcohol are preserved upon multilayer adsorption on the surface of CNMs which supports our prior findings on their separation performance. Upon exposure to the vaporous mixture, the membrane under investigation was found to pass heavy water with the transport rate of 35 kg m^−2^ h^−1^ and the selectivity of 330 meaning it did separate the two substances.^[11]^ Given the above comparison of the mixture with the pure vapors, the surface coverage seems to be of the same scale for heavy water, n‐propanol, and the azeotrope. It is likely that the sponge‐like membrane morphology offers specific domains for accumulation of the adsorbates and gives rise to their agglomeration so as the cluster size is somewhat limited. As the peak position for D_2_O remained unchanged, we believe the alcohol molecules to effectively replace water in the agglomerates.

Although quantifying the absolute number of adsorbed molecules in PM IRAS remains challenging, the data presented in Figure 3 can be viewed as qualitative adsorption isotherms that shed light on the strength of vapor‐membrane interactions. In particular, water vapor appears to readily condense on CNMs, and at humidity of 50 %, the uptake amounts to almost 70 % of that at saturation. The result suggests water molecules to rapidly cover the membrane surface in agreement with the permeation measurements and related kinetic simulations.^[9]^ Moreover, the transport model utilized before predicted water nanodroplets to be formed upon condensation which fits nicely to the spectroscopic picture obtained in the current study. Further efforts are foreseen on developing adequate calibration systems to relate PM IRAS signals with the real surface coverage. As the chemical character of 2D membranes varies much, it is likely to impact on adsorption energetics and the respective ACP rates. Potentially, one could convert the spectral intensity into the amount of adsorbates and obtain adsorption isotherms which would enable straightforward structure‐property correlations.

## Conclusions

The paper described PM IRAS measurements on the adsorption of vaporous substances upon supported 2D membranes. The experimental apparatus was devised for creating well‐defined environments that closely resemble operational parameters in functional tests. The system performance was demonstrated with CNMs exposed to heavy water, n‐propanol, and their binary azeotrope. The species were revealed to condense on the membrane surface depending on the applied feed pressure. With the help of a reference liquid‐phase experiment, we established the azeotrope to adsorb uniformly as a mixture of the same content. Both the pure vapors and their mixture were found to spread over the membrane with a similar degree of surface coverage. The spectroscopic evaluation was shown to serve as a powerful supplement to the permeation results on suspended layers and to firmly verify the concept of ACP in 2D membranes.

Understanding elementary physicochemical processes in conventional membranes has been slow compared to catalytic reactions, and only recently *operando* membrane characterization has begun coming to life.^[27]^ While the field of 2D membranes is still in its infancy, we aspire to get insights into mechanisms of the mass transfer and molecular separation by taking lessons from surface science and catalysis. Even though there are issues on determining the absolute surface coverage of adsorbates, the experimental approach implemented substantially expands the scope of 2D membranes research. This work is anticipated to be useful not only in rationalizing the permeation rates, but also for planning cutting‐edge experiments. For example, chiral separation with 2D membranes has been proposed that is based on surface modification and intermolecular complexation.^[28]^ In light of our findings, it seems feasible to test the idea by first studying the adsorption of enantiomers on functionalized membranes and then measuring the permselectivity.

## Experimental Section

The PM IRAS measurements were conducted with a FTIR spectrometer VERTEX 70 (Bruker) and a polarization modulation accessory PMA50 (Bruker). Both compartments were constantly purged with dry nitrogen at the flow rate of 3 L min^−1^. For each measurement, 500 scans were acquired with resolution of 4 cm^−1^. The spectra with liquid droplets were recorded with 200 scans. The Refractor^TM^ reactor was purchased from Harrick Scientific Products. The pressure was measured with a Baratron capacitance manometer (MKS Instruments), and the system was evacuated with a nXDS15iC scroll pump (Edwards). Heavy water (Sigma‐Aldrich, 99.9 % atom D), 1‐propanol (Chem Solute, 99.5 %), and their mixture were repeatedly degassed prior to experiments.

CNM was prepared by electron irradiation from *p*‐terphenyl‐4‐thiol (Sigma‐Aldrich, 97 %) assembled on an epitaxial Au(111)/mica substrate (Georg Albert PVD).^[4]^ The radiation‐induced cross‐linking of the aromatic molecules resulted in ∼1.2 nm thick carbonaceous films. The material structure was amorphous with a sponge‐like surface topography and tortuous nanochannels. The areal pore density was estimated to be greater than 10^13^ cm^−2^, and their size distribution was ranging from 0.3 to 0.7 nm. The CNM membrane on the native gold support was loaded into the Refractor^TM^ chamber without further treatment. The reactor was pumped out for a few hours before exposure to vapors.

## Conflict of interest

The authors declare no conflict of interest.

## References

[1a] F. Moghadam , H. B. Park , 2D Mater. 2019, 6, 042002;

[1b] L. Prozorovska , P. R. Kidambi , Adv. Mater. 2018, 30, 1801179. 10.1002/adma.201801179 30085371

[2] A. Schneemann , R. Dong , F. Schwotzer , H. Zhong , I. Senkovska , X. Feng , S. Kaskel , Chem. Sci. 2021, 12, 1600–1619. 10.1039/d0sc05889k PMC8179301 34163921

[3] C. Büchner , M. Heyde , Prog. Surf. Sci. 2017, 92, 341–374.

[4] P. Dementyev , D. Naberezhnyi , M. Westphal , M. Buck , A. Gölzhäuser , ChemPhysChem 2020, 21, 1006–1011. 32202365 10.1002/cphc.202000150 PMC7317367

[5a] S. W. Park , Z. Liao , B. Ibarlucea , H. Qi , H.-H. Lin , D. Becker , J. Melidonie , T. Zhang , H. Sahabudeen , L. Baraban , C.-K. Baek , Z. Zheng , E. Zschech , A. Fery , T. Heine , U. Kaiser , G. Cuniberti , R. Dong , X. Feng , Angew. Chem. Int. Ed. 2020, 59, 8218–8224; 10.1002/anie.201916595 PMC7317805 32039541

[5b] G. Hutchings , X. Shen , C. Zhou , P. Dementyev , D. Naberezhnyi , I. Ennen , A. Hütten , N. Doudin , J. Hsu , Z. Fishman , U. Schwarz , S. Hu , E. Altman , submitted to 2D Mater. .

[6] D. Naberezhnyi , S. W. Park , W. Li , M. Westphal , X. Feng , R. Dong , P. Dementyev , Small 2021, 2104392. 10.1002/smll.202104392 34713582

[7a] D. Naberezhnyi , P. Dementyev , Phys. Chem. Chem. Phys. 2020, 22, 9808–9814; 32337528 10.1039/d0cp01233e

[7b] W. Guo , S. M. Mahurin , R. R. Unocic , H. Luo , S. Dai , Nano Lett. 2020, 20, 7995–8000. 33064492 10.1021/acs.nanolett.0c02860

[8] P. L. Krapivsky , S. Redner , E. Ben–Naim , A Kinetic View of Statistical Physics, Cambridge University Press, Cambridge, 2010.

[9] P. Dementyev , T. Wilke , D. Naberezhnyi , D. Emmrich , A. Gölzhäuser , Phys. Chem. Chem. Phys. 2019, 21, 15471–15477. 31257369 10.1039/c9cp03038g

[10a] A. Singh , G. P. Rangaiah , Ind. Eng. Chem. Res. 2017, 56, 5147–5163;

[10b] L. M. Vane , J. Chem. Technol. Biotechnol. 2019, 94, 343–365. 30930521 10.1002/jctb.5839 PMC6436640

[11] P. Dementyev , Y. Yang , M. Rezvova , A. Gölzhäuser , J. Phys. Chem. Lett. 2020, 11, 238–242. 31846582 10.1021/acs.jpclett.9b03256

[12] M. Rezvova , A. Gölzhäuser , P. Dementyev , Adv. Mater. Interfaces 2020, 2000121.

[13] T. Buffeteau , B. Desbat , J. M. Turlet , Appl. Spectrosc. 1991, 45, 380–389.

[14] B. E. Hayden in Vibrational Spectroscopy of Molecules on Surface (Eds.: J. T. Yates , Jr., T. E. Madey ), Springer Science+Business Media New York, New York, 1987, pp. 267–344.

[15] T. Buffeteau , B. Desbat , D. Blaudez , J. M. Turlet , Appl. Spectrosc. 2000, 54, 1646–1650.

[16a] R. A. Dawe , D. M. T. Newsham , S. Bee Ng , J. Chem. Eng. Data 1973, 18, 44–49;

[16b] E. B. Munday , J. C. Mullins , D. D. Edle , J. Chem. Eng. Data 1980, 25, 191–194;

[16c] C. Gabaldon , P. Marzal , J. B. Monton , M. A. Rodrigo , J. Chem. Eng. Data 1996, 41, 1176–1180.

[17] L. De Marco , W. Carpenter , H. Liu , R. Biswas , J. M. Bowman , A. Tokmakoff , J. Phys. Chem. Lett. 2016, 7, 1769–1774. 27115316 10.1021/acs.jpclett.6b00668

[18] H. Belhadj , A. Hakki , P. K. J. Robertson , D. W. Bahnemann , Phys. Chem. Chem. Phys. 2015, 17, 22940–22946. 26266701 10.1039/c5cp03947a

[19] H. Ogasawara , J. Yoshinobu , M. Kawai , Chem. Phys. Lett. 1994, 231, 188–192.

[20] I. Engquist , I. Lundström , B. Liedberg , A. N. Parikh , D. L. Allara , J. Chem. Phys. 1997, 106, 3038–3048.

[21] S. G. Moussa , T. M. McIntire , M. Szὄri , M. Roeselová , D. J. Tobias , R. L. Grimm , J. C. Hemminger , B. J. Finlayson-Pitts , J. Phys. Chem. A 2009, 113, 2060–2069. 19173586 10.1021/jp808710n

[22] P. Cao , K. Xu , J. O. Varghese , J. R. Heath , Nano Lett. 2011, 11, 5581–5586. 22050080 10.1021/nl2036639

[23] Y. Shimazaki , I. Arakawa , K. Yamakawa , AIP Adv. 2018, 8, 045313.

[24] E. K. Plyler , J. Res. Natl. Bur. Stand. 1952, 48, 281–286.

[25] I. Doroshenko , V. Pogorelov , V. Sablinskas , Dataset Papers in Science 2013, 2013, Article ID 329406, 6 pages.

[26] S. Brunauer , P. H. Emmett , E. Teller , J. Am. Chem. Soc. 1938, 60, 309–319.

[27] C. P. O'Brien , J. Membr. Sci. 2021, 619, 118751.

[28] S. M. Fruehwirth , R. Meyer , A. W. Hauser , ChemPhysChem 2018, 19, 2331–2339. 29863766 10.1002/cphc.201800413 PMC6175349

